# Racial Disparities in Sleep Among Diverse Young Adults During the First Semester of College

**DOI:** 10.1007/s40615-025-02592-6

**Published:** 2025-08-18

**Authors:** Tiffany Yip, Kyle Lorenzo, Jiawei Wu, Jinjin Yan, Zhenqiang Zhao, Heining Cham, David Chae, Mona El‑Sheikh

**Affiliations:** 1 Department of Psychology, Fordham University, 441 East Fordham Road, Bronx, NY 10458, USA; 2 Department of Social, Behavioral, and Population Sciences, Tulane University, New Orleans, USA; 3 Department of Human Development and Family Studies, Auburn University, Auburn, USA

**Keywords:** Sleep, Young adults, College transition, College students

## Abstract

This study investigated patterns of disparities in sleep during the first semester of college, focusing on several dimensions of sleep and using multiple methods. Ethnically and racially diverse first-year college students (n = 635; Asian = 20%, Black = 12%, Latinx = 20%, multiracial = 23%, White = 25%; female = 73%, male = 25%, non-binary = 2%) completed a baseline survey, 14 daily diaries, and wore wrist actigraphs in their first semester at college. Across all three methodologies, White students had the longest sleep duration, whereas Black students had the shortest. Across surveys and daily diaries, White students reported the most environmental sleep disturbances. Asian students experienced the poorest actigraphy-assessed sleep efficiency and greatest wake minutes after sleep onset, despite reporting similar levels of sleep quality to other groups. These findings document ethnic/racial sleep disparities among diverse college students across a range of sleep dimensions and offer insight into developing focused institution-level interventions to target specific sleep outcomes.

## Introduction

The first semester of college represents a dramatic change for emerging adults (18–29 years old) [1]. Exposure to new stressors such as adjusting to new social environments, greater academic demands, new living arrangements, and meeting new people can drive these changes. To minimize the negative impact of these stressors on college student adjustment, there is increasing interest in how stress exposures are related to the sleep health of college students [1–3]. Sleep health refers to the multidimensional nature of sleep–wake patterns that is linked with physical and mental well-being, as well as academic performance [4–6]. Sleep disturbances are prevalent during college and have far-reaching public health implications. Research on sleep among college students has found that over 60% of the 1,125 college students report poorer sleep quality [7]. Similar rates have been observed in other samples, where 62% of 7,626 college students met the criteria for poor sleep [8]. This prevalence of poor sleep among college students is particularly concerning because poor sleep is associated with compromised physical and psychological health [9]. A recent meta-analysis on college student sleep by Gardani et al. (2022) found associations between sleep quality and stress (*r* = 0.39, n = 25 studies) as well as between insomnia and stress (*r* = 0.41, n = 12 studies) [10]. However, more research is needed to explore disparities in sleep among emerging adults during the first semester of college, or if these disparities vary across measurements of sleep.

Sleep is a multidimensional health behavior [11] encompassing a range of activities such as bedtime behaviors, physiological processes, sleep–wake transitions, and daytime outcomes [12]. Therefore, examining various measures of sleep is imperative to gain a more holistic and accurate understanding of sleep health [11, 13]. Capturing multiple dimensions of sleep not only sheds light on nuanced patterns of sleep disparities but also helps identify specific areas of need for diverse college students to inform efforts focused on improving sleep health [9]. This study examines bedtime factors (bedtime, rise time, total time in bed), sleep parameters (duration, quality, latency, efficiency, wake after sleep onset), environmental characteristics (disturbances), and daytime behavior (dysfunction).

## Ethnic/Racial Disparities in Sleep

Although sleep health may be compromised for all students during college, ongoing research has highlighted disparities in sleep across sociodemographic characteristics such as ethnicity and race [14–16]. Therefore, in order to fully understand sleep during college, it is important to systematically consider disparities related to ethnicity and race. Specifically, ethnically and racially minoritized students are more likely to report short sleep and lower sleep quality than their White counterparts in the first month of college [17]. These disparities continue throughout college with White students reporting more favorable sleep compared to ethnically/racially minoritized students. A recent study found that relative to White students, Black college students reported poorer sleep outcomes, including shorter sleep duration and poorer sleep quality [18]. Using nationally representative survey data from over 2,000 college students, Jones et al. (2020) found a significant difference in short sleep duration such that White college students reported a lower likelihood of short sleep compared to Black, but not Asian or Latinx, college students [19]. Gaultney (2010) found that Black and Asian college students report less risk for insomnia and fewer poor sleep practices compared to White and Latinx students [20]. Finally, Goodin et al. (2010) found no significant differences in sleep quality among a college sample of Asian, Black, and White students [21]. While there is some research observing disparities between White and students of color, it is less clear if there are disparity patterns between ethnically and racially minoritized young people. Further research is needed to investigate ethnic/racial disparities among college students, especially as the diversity of the United States college population continues to grow [22]. In addition, there is a need to examine multiple dimensions of sleep across different measures when investigating ethnic/racial disparities. Due to the long-term benefits of sleep for health and academic success, examining these ethnic/racial disparities during the first semester of college can provide insight into how college impacts sleep differently across college students from diverse backgrounds [9].

## Capturing Sleep Health Using Multiple Methodologies: Surveys, Daily Diaries, and Wrist Actigraphs

Comprehensive studies of sleep health often employ various validated measures to capture multiple domains of sleep [11, 13, 23]. In order to obtain a more comprehensive understanding of disparities in sleep, it is important to assess multiple dimensions of sleep across a range of methods. In the current study, we include retrospective surveys, daily diaries, and wrist actigraphs. The strengths and limitations of each of these methods are discussed below.

Surveys often include standardized scales, such as the Pittsburgh Sleep Quality Index [24], which have been found to be valid and reliable. Survey measures capture information about individuals’ perceptions of their sleep patterns, quality, and disturbances, and can be modified for a particular time frame (i.e., *over the past month, in the past two weeks, in general*) [24, 25]. Survey measures have the benefit of being easy to administer and requiring only one report to summarize sleep patterns for a specified timeframe. This convenience makes surveys ideal for sleep health across large populations [25]. At the same time, surveys can also be subject to reporting biases by relying on participants’ retrospective recall of their sleep behaviors [25].

In contrast, daily diary methodologies capture dynamic sleep on a day-to-day basis [16, 26]. This approach involves participants reporting their sleep patterns and behaviors daily across multiple, consecutive days, providing a more accurate record of same-day sleep. In addition, having multiple reports of sleep captures dynamic sleep behaviors and patterns. For example, two-week daily diary studies offer the ability to not only investigate mean levels of sleep over time but also a systematic analysis of day-to-day variability in sleep [11, 16]. Therefore, daily diary methodologies can enhance the accuracy of reported sleep patterns and allow for more complex analyses [27]. However, because they place more burden on respondents, daily diary designs are typically limited to shorter timeframes. Measuring sleep using both surveys and daily diary designs can offer complementary insights into sleep behaviors, capturing both broader perceptions of sleep over time as well as day-to-day perceptions [28]. Incorporating both can ultimately serve to create a nuanced depiction of sleep experiences that more accurately reflects the complex nature of sleep among college students.

Finally, wrist actigraphy provides an ecologically valid, objective measure of sleep that tracks movement to estimate sleep and wake periods [29, 30]. Wrist actigraphs allow researchers to monitor sleep continuously over extended periods. Another benefit of wrist actigraphy is that it provides real-time data on sleep duration, variability, and disruptions with greater accuracy [31]. However, actigraph devices primarily rely on sleep based on movement, which may result in discrepancies between wakefulness and motionless wake states, especially if individuals engage in quiet activities before bed [32]. In addition, although actigraphy can provide more real-time sleep data, the accuracy of the data depends on participant compliance in wearing the device consistently. As such, technical issues with the device or removal of the watch can lead to missing or unreliable data [30].

## The Current Study

Despite growing concerns about sleep health among United States college students, there is less data on ethnic/racial sleep disparities during the first semester of college [33]. This requires collecting multiple indicators of sleep among ethnically and racially diverse college students. The current study uses a multi-method approach including surveys, daily diaries, and wrist actigraphs to investigate ethnic/racial sleep disparities during the first semester of college among a large sample of ethnically and racially diverse students. Based on prior findings [15], it is hypothesized that White college students will experience better sleep health overall (e.g., longer sleep duration, better sleep quality, healthy range of sleep onset latency, etc.) across all methods of data collection compared to all other ethnic/racial groups. Additionally, it is hypothesized that Black college students will experience poorer sleep health (e.g., shorter sleep duration, poorer sleep quality [34]) compared to all other groups. However, given the mixed and limited findings of disparities between Asian, Latinx, and multiracial college students [19, 20], these analyses are exploratory.

## Methodology

### Sample

This paper presents the first year, first semester data from an ongoing 5-year, longitudinal, IRB-approved, sleep study of college students at a private, predominantly White private University in a metropolitan city in the northeastern United States. The current sample consists of 635 first-year, first-semester college students (Asian = 20%, Black = 12%, Latinx = 20%, multiracial = 23%, White = 25%; female = 73%, male = 25%, non-binary = 2%). Of the multiracial students, the most common ethnic/racial groups represented were Latinx and White (n = 47), Latinx and Black (n = 32), and Asian and White (n = 21). Other groups included Asian and Black (n = 5), Asian and Latinx (n = 8), and Black and White (n = 8). The mean age of the sample was 18.44 years old (*SD* = 0.44). Over one-third of the sample (38.2%) were from the same state before entering college, and about another one-third came from urban areas (30%). The most frequent states students came from were New Jersey (14%), Connecticut (6%), California (5%), and Massachusetts (5%). A primary aim of the project was to investigate ethnic/racial disparities in sleep.

### Procedure

First-year students were recruited through school emails, physical flyers, and in-person tabling. Students were told that they could earn compensation for participating in a longitudinal sleep study for their four years in college. Prospective participants provided informed consent at the beginning of an online screener form. The screener determined eligibility which included: first-semester, first-year undergraduate student, without a medically-diagnosed sleep disorder, asthma, or chronic health condition that would interfere with normative sleep processes. Similarly, international students were ineligible due to possible effects of jetlag.

Data collection occurred for the entire fall semester of the academic year (August – December) for three cohorts of undergraduate classes (Cohort 1 = fall 2021, Cohort 2 = fall 2022, Cohort 3 = fall 2023). Eligible participants completed an online survey (average response time = 32.40 min; *SD* = 10.20 min), where students reported on their demographics, stressors, sleep, health, and academics. Data were de-identified. After completing this survey, participants were presented with a link to an online scheduling website for an in-person lab appointment to pick up a wrist actigraph and begin the 14-day daily diary procedure. Participants were given the option to receive their daily diaries via text or email (text = 99%, email = 1%). Each night, participants completed a 5- to 10-min online survey (average response time = 7.53 min; *SD* = 1.37 min), where they reported on their everyday stressors, sleep, mood, and well-being before bed for 14 consecutive nights (*M* = 13.30 completed days; *SD* = 3.66 completed days). All online surveys were distributed via Qualtrics. Based on feedback from college students and their typical schedules, nightly surveys were sent at 7:30 PM every night. If by 11:00 PM, participants had not yet completed their survey for the day, a reminder message was sent. Because many students reported staying up late to study, participants had until 5:00 AM of the following morning to complete the survey about their sleep the prior night. If a participant failed to complete the nightly survey, a member of the research team messaged them. In the event that a respondent missed more than one night, a member of the research time called them. Each participating student was compensated (e.g., $25 for completing the survey and up to $28 for completing the daily diaries). At the end of the 14 days, participants dropped off their sleep watch.

## Measures

### Ethnicity and Race

Ethnicity and race were categorized into 5 distinct groups (i.e., Asian, Black, Latinx, multiracial, & White) using self-reported primary race, secondary race, and university records. Participants responded to the prompt, *“What do you consider to be your PRIMARY race?*”, with responses (0 = American Indian/Native American or Alaska Native; 1 = Asian or Asian American; 2 = Black, African American, or West Indian; 3 = Hispanic or Latinx; 4 = Middle Eastern or North African; 5 = Native Hawaiian or other Pacific Islander; 6 = White; and 7 = Not Listed). Participants also responded to the prompt, “*Do you consider yourself to be a member of MORE THAN ONE racial group?”* (0 = No, 1 = Yes), followed by identical response options if they selected “Yes”.

University records for ethnicity and race consisted of six categories: White, Black, Hispanic/Latinx, Asian, American Indian/Alaska Native, and Two or More Races. Ethnicity and race were cross-referenced between self-report and university records to create a total of five categories. Of note, because the grant was focused on recruiting Asian, Black, Latinx, White, and multiracial students specifically, participants were assigned to one of these 5 groups. Participants in the “multiracial” group were those who reported more than one ethnicity and race (e.g., reporting both White & Latinx or reporting Black, Asian, and Latinx). In addition, instances where ethnic and racial categories differed (e.g., participants self-reported “Hispanic or Latinx” but were categorized as “Black” in university records) were categorized as “multiracial”.

### Covariates

Gender and Pell recipient status were included as covariates in analyses. Gender was captured by asking participants to respond to the prompt, *“What is your gender”* with 5 options (0 = *Male*, 1 = *Female*, 2 = *Non-binary*, 3 = *Prefer not to say*, and 4 = *Not listed*). Those who responded “Not listed*”* were given an option to submit an open-ended response. The current sample consisted of 73% female, 25% male, and 2% non-binary. No participants selected “Prefer not to say” or “Not listed”.

Pell grant recipient was used as a measure for socioeconomic background and collected using university records. Pell recipient status was coded as 0 (*Did not receive Pell*) or 1 (*Pell recipient*), with a score of 1 indicating lower socioeconomic background. The current sample consisted of 61% participants who were Pell recipients and 39% of participants who did not receive the Pell.

### Survey Measures of Sleep

The Pittsburgh Sleep Quality Index [24] was used to assess sleep latency, duration, quality, bedtime, rise time, total time in bed, and daytime dysfunction in the past month. The Sleep Environment Inventory [35] was used to assess sleep disturbances since the start of the fall semester.

#### Sleep Latency

Participants reported how long it typically took them to fall asleep, *“During the past month, how long (in minutes) has it usually taken you to fall asleep each night?”.* Responses were captured on a 4-point scale and coded so that higher values indicated more time it took to fall asleep (0 = *less than 15 min*, 1 = *16–30 min*, 2 = *31–60 min*, 3 = *more than an hour*). The mean sleep latency for the current sample was 0.88 (*SD* = 0.83), equivalent to responses between “Less than 15 min” and “16–30 min”.

#### Sleep Duration

Participants reported their typical sleep duration,*“During the past month, how many hours of actual sleep did you get at night? (This may be different than the number of hours you spent in bed)”.* Responses were captured on a 4-point scale and coded so that higher values indicated more hours of sleep (0 = *less than 5 h*, 1 = *5–6 h*, 2 = *6–7 h*, 3 = *greater than 7 h*). The mean sleep duration for the current sample was 1.74 (*SD* = 0.83), which is equivalent to between “5–6 h” and “6–7 h”.

#### Sleep Quality

Participants rated their overall sleep quality,*“*During the past month, how would you rate your overall sleep quality?”. Responses were captured on a 4-point scale (0 = *very good*, 1 = *fairly good*, 2 = *fairly bad*, 3 = *very bad*). This item was reverse-scored, with the higher scores indicating higher sleep quality. The mean sleep quality for the current sample was 1.89 (*SD* = 0.56), which is equivalent to between “Fairly Bad” and “Fairly Good”.

#### Bedtime

Participants reported the time they typically went to bed by selecting the hour (0 = *7 PM* to 11 = *6 AM*) and minute (in 5-min intervals, ranging from 0 = *0 min* to 11 = *55 min*). Bedtimes were converted into numeric values representing minutes in reference to midnight (e.g., 12:30 AM → 30; 10:00 PM → −120). The mean bedtime for the current sample was 12:25 AM (*SD* = 1 h and 14 min).

#### Rise Time

Participants reported the time they typically got up by selecting the hour (0 = *4 AM* to 14 = *6 PM*) and minute (in 5-min intervals, ranging from 0 = *0 min* to 11 = *55 min*). Rise times were converted into numeric values with midnight as the reference point (e.g., 7:40 AM → 460). The mean rise time for the current sample was 8:03 AM (*SD* = 1 h and 19 min).

#### Total Time in Bed

Total time in bed was calculated as the difference between bedtime and rise time. The mean total time in bed for the current sample was 7 h and 35 min (*SD* = 1 h and 25 min).

#### Daytime Dysfunction

Participants answered two questions about daytime dysfunction: (1) “During the past month, how often have you had trouble staying awake while doing homework, while in class, eating meals, or spending time with friends?”. This item was rated on a 4-point scale (0 = *not during the past month*, 1 = *less than once a week*, 2 = *once or twice a week*, 3 = *three or more times a week*); and (2) “During the past month, how much of a problem has it been for you to keep up enough enthusiasm (e.g., energy) to get things done?” This item was rated on a 4-point scale (0 = *not a problem at all*, 1 = *only a very slight problem*, 2 = *somewhat of a problem*, 3 = *a very big problem*). The scores of the two items were summed, with higher scores indicating greater daytime dysfunction. The mean daytime dysfunction score for the current sample was 2.81 (*SD* = 1.53), indicating moderate daytime dysfunction.

#### Sleep Environment

The Sleep Environment Inventory (SEI) was used to assess for factors that commonly disrupt sleep [36]. Participants were provided with seven items representing environmental disturbances (e.g., “noise outside,” “TV, radio, or computer”) and asked to report whether each item kept them from sleeping well since the start of this academic year, by indicating yes (1) or no (0). The scores of the seven items were summed, with higher scores indicating more environmental sleep disturbances. The mean sleep environment score for the current sample was 2.20 (*SD* = 1.59), corresponding to about two environmental disturbances.

### Daily Diary Measures of Sleep

The daily diaries administered a modified, daily version of the PSQI to assess bedtime, rise time, total time in bed, sleep latency, duration, quality, and daytime dysfunction (e.g., *last night, this morning, today*). The Sleep Environment Inventory was used to assess sleep disturbances [35].

#### Daily Sleep Latency

Participants reported how long it took them to fall asleep *last night* (0 = *0–5 min*, 1 = *6–10 min*, 2 = *11–15 min*, 3 = *16* + *minutes*, 4 = *Did not go to sleep*). The mean daily sleep latency for the current sample was 1.25 (*SD* = 1.07), which is equivalent to between “6–10 min” and “11–15 min”.

#### Daily Sleep Duration

Participants reported how many hours of actual sleep they got *last night* (0 = *0–1 h*, 1 = *2 h* … 11 = *12* + *hours*). The mean sleep duration for the current sample was 6.07 (*SD* = 1.75), which corresponds to a little over “5 h”.

#### Daily Sleep Quality

Participants rated their overall sleep quality *last night* on a 4-point scale (0 = *very bad*, 1 = *fairly bad*, 2 = *fairly good*, 3 = *very good*). The mean daily sleep quality for the current sample was 2.02 (*SD* = *0.7*4), which corresponds to “Fairly Good”.

#### Daily Bedtime

Participants reported the time they went to bed *last night*, by selecting the hour (0 = *12 AM* to 23 = *11 PM*) and minute (in 15-min intervals, ranging from 0 = *0 min* to 3 = *45 min*). Bedtimes were converted into numeric values representing minutes in reference to midnight (e.g., 12:30 AM → 30; 10:00 PM → −120). The mean daily bedtime for the current sample was 1:14 AM (*SD* = 4 h and 11 min).

#### Daily Rise Time

Participants reported the time they woke up *this morning*, by selecting the hour (0 = *12 AM* to 23 = *11 PM*) and minute (in 15-min intervals, ranging from 0 = *0 min* to 3 = *45 min*). For analysis purposes, rise times were converted into numeric values with midnight as the reference point (e.g., 7:40 AM → 460). The mean daily rise time for the current sample was 8:38 AM (*SD* = 2 h and 10 min).

#### Daily Total Time in Bed

Total time in bed was calculated as the difference between bedtime and rise time. The mean total time in bed for the current sample was 8 h and 54 min (*SD* = 4 h and 35 min).

#### Daily Sleep Environment

Participants were provided with seven items (e.g., “noise outside,” “TV, radio, or computer”) and asked to report whether each item kept them from sleeping well *last night*, by indicating yes (1) or no (0). The seven items were summed, with higher scores indicating more sleep disturbances in the environment. The mean daily sleep environment score for the current sample was 0.65 (*SD* = 1.06), falling between zero to one environmental disturbance.

#### Daily Daytime Dysfunction

Participants answered two questions (i.e., “Today, did you have trouble staying awake while studying, eating meals, or engaging in social activity?” “Today, did you have problems keeping up enthusiasm to get things done?”) by indicating yes (1) or no (0). The scores of the two items were summed, with higher scores indicating greater daytime dysfunction. The mean daytime dysfunction score for the current sample was 0.54 (*SD* = 0.76), indicating low levels of daytime dysfunction.

### Wrist Actigraphy Measures of Sleep

Sleep data were collected using the Philips Actiware wrist actigraph for 14 consecutive nights [37]. Wrist actigraphs detect sleep and wake patterns and were configured to capture 1-min epochs using Sadeh’s algorithm, with the wake threshold set to 40 activity counts [38]. Data were retrieved and sleep periods were coded by research assistants and corroborated with self-reported sleep and wake times from the daily diary. Research assistants coded the same five practice files to achieve interrater reliability (IRR) using intraclass correlations. Discrepancies were discussed and files were re-coded until there was good IRR (intraclass correlations = 0.89–0.96) for these practice files. Once a coder achieved reliability, they were permitted to code files independently and every 10th file was double coded to prevent drift throughout the coding process. Files were reliably coded with a mean IRR of 0.94.

#### Sleep Onset

Sleep onset refers to the time that a participant falls asleep. This score was automatically detected by the actigraph based on the coded bedtime. The mean actigraph sleep onset for the sample was 1:24 AM (*SD* = 1 h and 52 min).

#### Sleep Offset

Sleep offset refers to the time that a participant wakes up. This score was automatically detected by the actigraph based on the coded rise time. The mean actigraph sleep offset for the sample was 8:45 AM (*SD* = 2 h and 0 min).

#### Sleep Duration

Sleep duration refers to the total number of minutes between an individual falling asleep and waking up. The mean actigraph sleep duration for the sample was 7 h and 20 min (*SD* = 1 h and 56 min).

#### Sleep Efficiency

Sleep efficiency was calculated as the proportion of minutes (0–100%) spent in sleep divided by total time in bed. The mean actigraph sleep efficiency for the sample was 82.14% (*SD* = 7.57%).

#### Wake after Sleep Onset (WASO)

Wake after sleep onset (WASO) was calculated as the number of wake epochs after falling asleep. The mean actigraph WASO for the sample was 46.42 min (*SD* = 26.32 min).

#### Sleep Inertia

Sleep inertia was measured as the number of minutes between sleep offset and the end of their rest period. The mean actigraph sleep inertia for the sample was 19.25 min (*SD* = 22.59 min).

#### Bedtime

Bedtime was calculated as the time when a participant entered a rest period, indicated as the time after three consecutive epochs with a wake activity level of 3 digits or more. The mean actigraph bedtime for the sample was 1:03 AM (*SD* = 1 h and 54 min).

#### Rise Time

Rise time was calculated as the time the participant emerged from a rest period, the time before three consecutive epochs with a wake activity level of 3 digits or more. The mean actigraph rise time for the sample was 9:04 AM (*SD* = 2 h and 2 min).

#### Sleep Latency

Sleep latency was measured as the number of minutes between a participant’s bedtime and the beginning of their sleep period. The mean actigraph sleep latency for the sample was 20.58 min (*SD* = 25.26 min).

## Analysis Plan

Analyses were conducted in RStudio (ver. 2024.4.2.764, R Core Team) [39]. The aim of this study was to examine differences in sleep across ethnic/racial groups (i.e., Asian, Black, Latinx, multiracial, White). Consistent with prior research examining ethnic/racial disparities in sleep [40]), one-way analysis of variance (ANOVA) tests were conducted for each sleep outcome (i.e., bedtime, rise time, total time in bed, sleep latency, sleep duration, sleep quality, sleep environment, and daytime dysfunction) with ethnicity and race as the grouping variable. Welch’s correction was applied to both the one-way ANOVA and pairwise comparisons to account for heterogeneity of variance across groups [41]. An F-test with a significance value of *p* < 0.05 indicated a significant difference in sleep between ethnic/racial group members, and a post-hoc pairwise comparisons were conducted to further identify specific differences between the five ethnic/racial groups [40]. Pairwise comparisons with a *p* < 0.05 indicated significant differences in a given sleep outcome between those two ethnic/racial groups.

Due to the repeated measures nature of the daily diary and actigraph data, linear mixed-effects models (LMM) using restricted maximum likelihood estimation in the ‘*lme4’* package were conducted to account for person-level variability [42]. Sleep measures were treated as the outcome variable with ethnic/racial group as a fixed effect, while including participants as a random effect to account for individual differences. Likelihood ratio tests were conducted to compare models with ethnicity and race as a grouping variable with baseline models without a grouping variable. A significant chi-square statistic with *p* < 0.05 indicated that sleep variables were significantly different across ethnic/racial groups.

Survey and daily diary data had identical sleep measures, resulting in 8 sleep dimensions for each method (i.e., bedtime, rise time, total time in bed, sleep latency, sleep duration, sleep quality, sleep environment, and daytime dysfunction). Actigraph sleep outcomes included some common outcomes (i.e., bedtime, rise time, sleep latency, sleep duration) as well as measures unique to actigraphy (i.e., sleep onset, sleep offset, sleep efficiency, wake after sleep onset, sleep inertia), resulting in 9 dimensions for actigraphy.

## Results

Table 1 and Table 2 presents descriptive results from one-way ANOVAs and t-tests examining differences in sleep by covariates (gender, SES). Across all three methodologies, non-binary participants had the poorest sleep outcomes, with males having the most optimal sleep. Female participants consistently had poorer sleep than males, but better sleep than non-binary participants. However, it is worth noting that the sample for non-binary participants is small (n = 13, or 2% of the sample). When examining, socioeconomic status, participants who were Pell grant recipients (indicating a lower socioeconomic background) had consistently poorer sleep than those who did not receive the Pell grant. This pattern was consistent across all three methodologies.

**Fig. 1:**
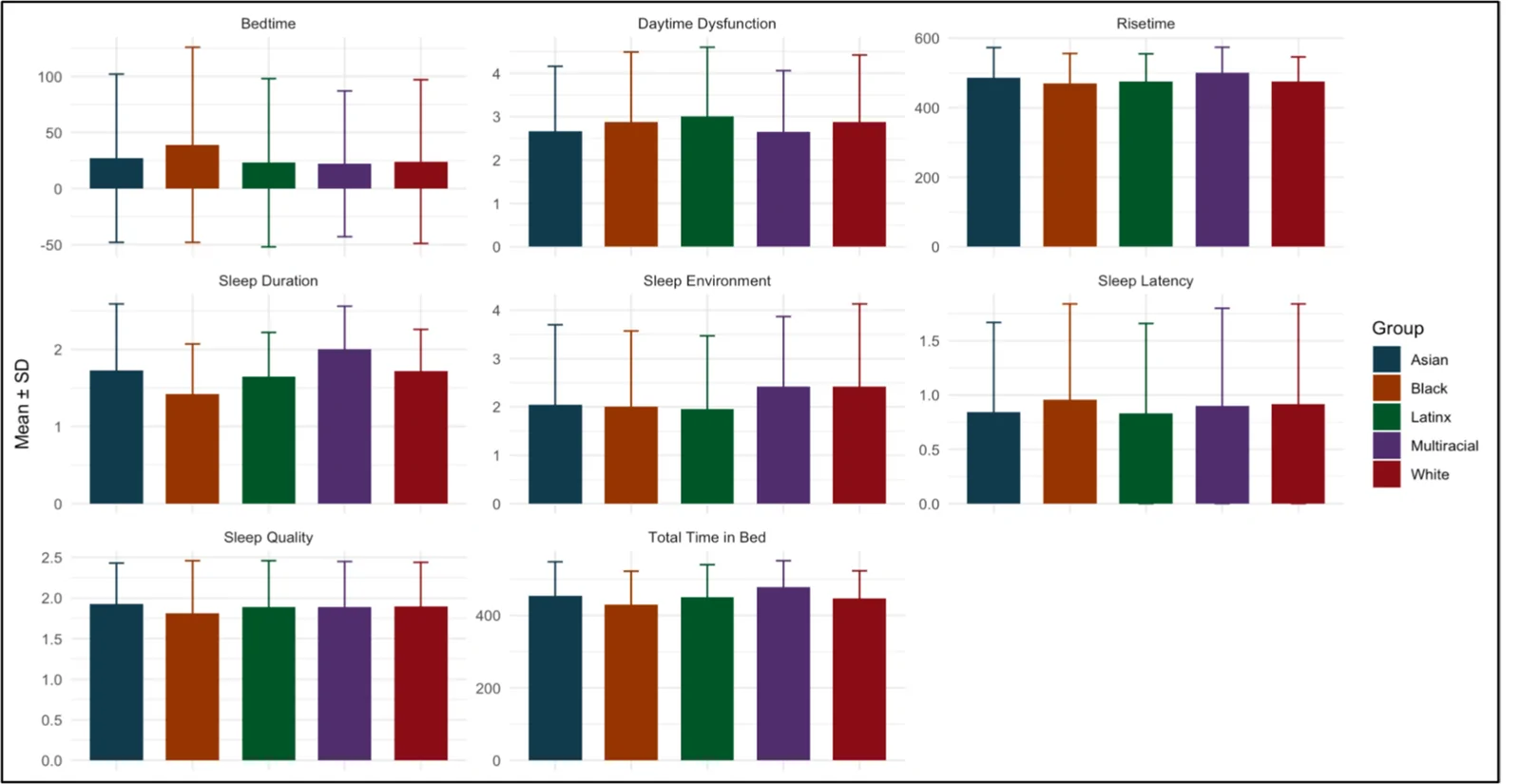
Ethnic/racial disparities in sleep: Survey. Values for bedtime and rise time represent minutes relative to midnight (0 = midnight)

**Fig. 2:**
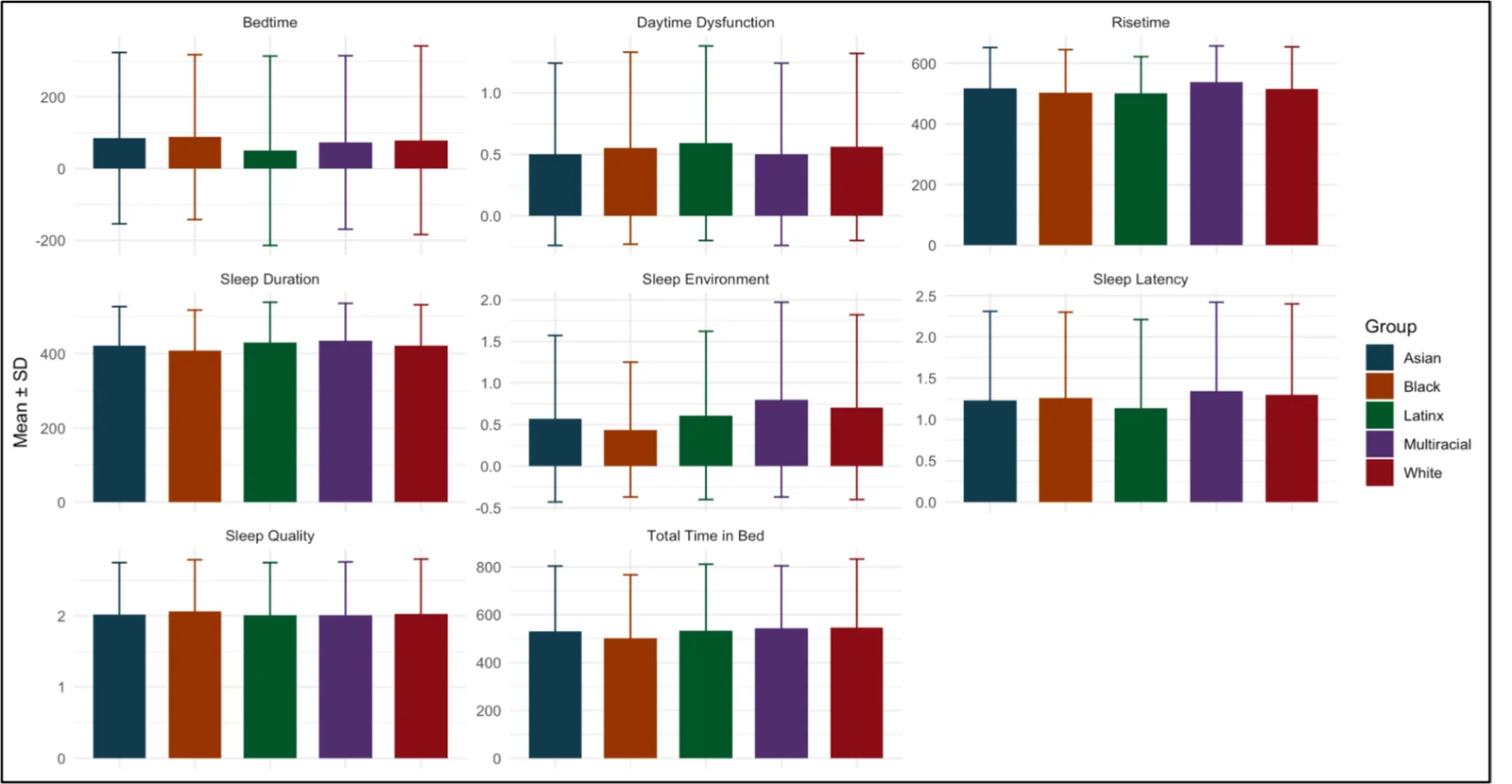
Ethnic/racial disparities in sleep: Daily diary. Values for bedtime and rise time represent minutes relative to midnight (0 = midnight)

**Fig. 3:**
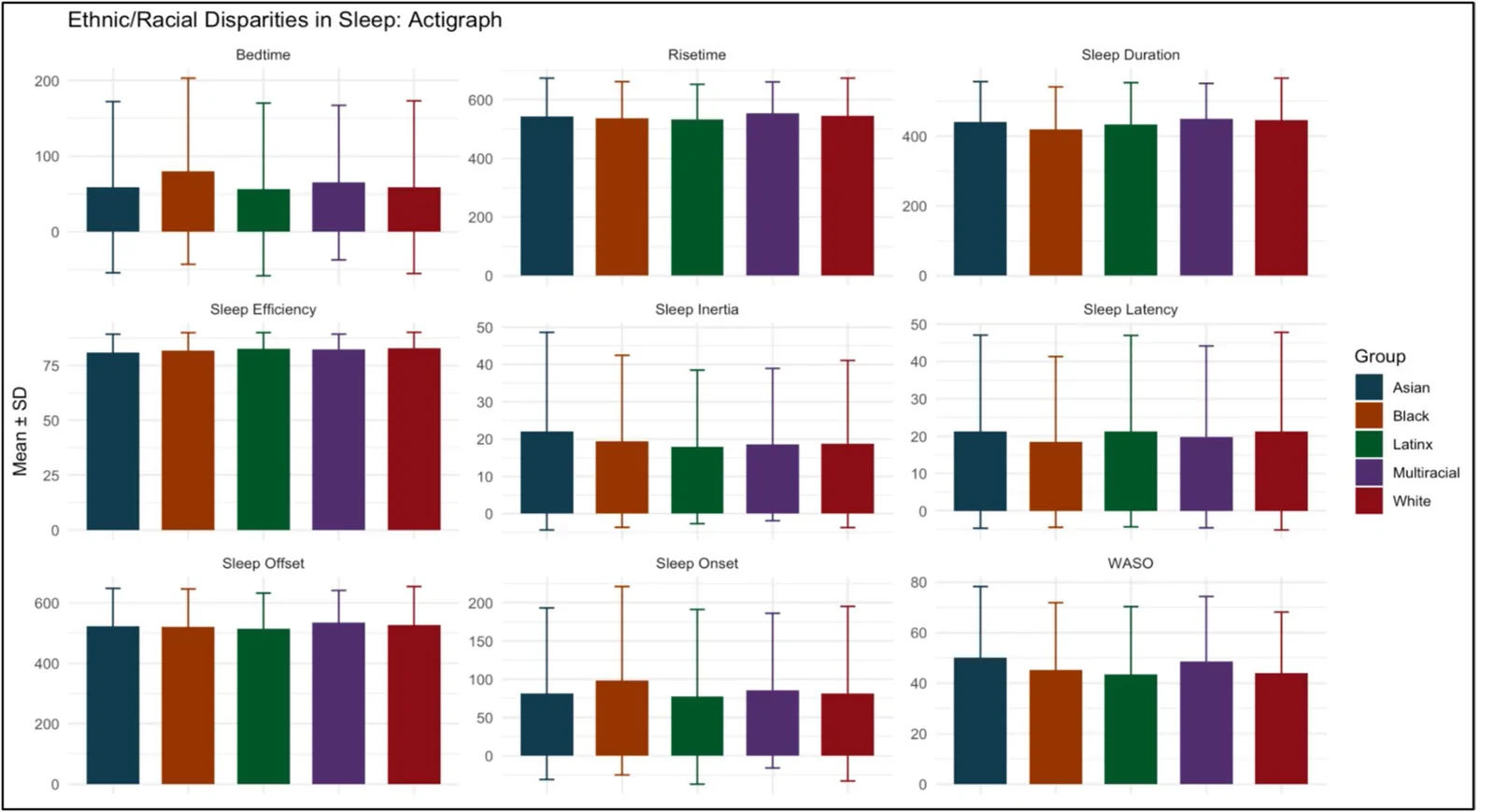
Ethnic/racial disparities in sleep: Actigraph. Values for bedtime, rise time, sleep onset, and sleep offset represent minutes relative to midnight (0 = midnight)

Table 3 presents results from all one-way ANOVAs and linear mixed-effects models examining ethnic/racial differences in sleep. Figures 1, 2 and 3 depict ethnic/racial group means of all sleep dimensions across the survey, daily diary, and actigraph data, respectively.

## Sleep Characteristics (Sleep onset, Sleep offset, Duration, Latency, Quality, Efficiency, WASO)

There were significant differences in sleep onset based on actigraphy data. Black students fell asleep significantly later than all other ethnic/racial groups (*M* = 1:38 AM, *SD* = 2 h and 3 min). There were no significant differences in wake time. Although not significant, White students woke up at the latest time (*M* = 8:55 AM, *SD* = 1 h and 46 min) and Latinx students woke up at the earliest time (*M* = 8:34 AM, *SD* = 1 h and 57 min).

Survey results revealed significant differences between ethnic/racial groups. White students reported significantly longer sleep duration (*M* = 2.00, *SD* = 0.77), corresponding to “6–7 h”, relative to all other groups. Multiracial and Asian students reported the second longest (*M* = 1.72—1.73, *SD* = 0.77—0.86) duration and were not different from each other, but both were significantly greater than Latinx students (*M* = 1.65*, SD* = 0.86), and Black students (*M* = 1.42, *SD* = 0.83) falling between “5–6 h” and “6–7 h”. Finally, Latinx students reported significantly longer sleep duration relative to Black students. Daily diary and actigraph data showed a similar pattern. White participants had significantly more daily reported sleep duration (*M* = 7 h and 14 min, *SD* = 1 h and 41 min) than Black students (*M* = 6 h and 49 min, *SD* = 1 h and 48 min). The actigraph data also showed significant differences in sleep duration between all ethnic/racial groups (*M* = 7 h and 13 min—7 h and 19 min, *SD* = 1 h and 42 min—2 h and 1 min) compared to Black students (*M* = 7 h and 1 min, *SD* = 2 h and 1 min).

There were significant differences in sleep latency based on survey and actigraphy responses. Based on actigraphy, Black students fell asleep significantly faster (*M* = 18.18 min, *SD* = 22.86) than all other ethnic/racial groups. White students fell asleep significantly faster (*M* = 19.43 min, *SD* = 24.35) than multiracial (*M* = 21.32 min, *SD* = 26.46) and Latinx students (21.18 min, *SD* = 25.63), but significantly slower than Black students. Finally, Asian students fell asleep significantly faster than Latinx students but significantly slower than Black students. These patterns diverged from the survey-reported data. According to the self-reported survey, Asian students fell asleep significantly faster (*M* = 0.84, *SD* = 0.83) compared to multiracial, White, and Black students (*M* = 0.90—0.96, *SD* = 0.81—0.88). Moreover, Latinx students fell asleep faster (*M* = 0.83, *SD* = 0.82) compared to multiracial students (*M* = 0.92, *SD* = 0.85). There were no significant differences in sleep latency for the daily diary data.

Sleep efficiency, WASO (wake after sleep onset), and sleep inertia were only collected by actigraphy. Asian students had significantly less sleep efficiency (*M* = 80.97%, *SD* = 8.31) compared to Latinx, multiracial, and White students (*M* = 82.32—82.77%, *SD* = 6.95—7.36). Specifically, Asian students had significantly more WASO (*M* = 49.71 min, *SD* = 28.29) compared to Black, Latinx, and multiracial students (*M* = 43.27—44.88 min, *SD* = 24.19—26.75). Asian students also experienced significantly greater sleep inertia (longer time to get out of bed after waking up; *M* = 22.12, *SD* = 26.51) compared to Latinx, multiracial, and White students (*M* = 17.89—18.67, *SD* = 20.42—20.60). As for self-reported sleep quality, a different pattern emerged from the actigraphy data. Survey data suggest Black students reported significantly lower levels of sleep quality (*M* = 1.81, *SD* = 0.65) relative to all other ethnic/racial groups (*M* = 1.89—1.93, *SD* = 0.50—0.57), although similar patterns were not observed for the daily diary reports.

## Bedtime Characteristics (Bedtime, Rise time, Total time in bed)

Baseline survey results indicated that Black students reported significantly later bedtimes (*M* = 12:39 AM, *SD* = 1 h and 27 min) than all other ethnic/racial groups (*M* = 12:22 AM—12:27 AM, *SD* = 1 h and 5 min—1 h and 15 min). Actigraphy data revealed a consistent pattern, with Black students also experiencing a significantly later bedtime (*M* = 1:20 AM, *SD* = 2 h and 3 min) than all other ethnic/racial groups (*M* = 12:56 AM—1:05 AM, *SD* = 1 h and 42 min—1 h and 54 min). However, no significant ethnic/racial effects emerged from the daily diary.

White students reported the latest survey rise times (*M* = 8:20 AM, *SD* = 1 h and 14 min), significantly later than all other ethnic/racial groups. Asian students reported the second latest survey rise time (*M* = 7:50 AM, *SD* = 1 h and 26 min), significantly earlier than White students but significantly later than Black, Latinx, and multiracial students. No significant differences in survey rise times emerged between Black, Latinx, and multiracial students. This pattern was consistent with the daily diary data, with White participants reporting significantly later daily rise times (*M* = 8:59 AM, *SD* = 1 h and 58 min) compared to Black, Latinx, and multiracial students (*M* = 8:22—8:35 AM, *SD* = 2 h and 0 min—2 h and 22 min). Actigraph results did not reveal significant differences, although White students still had the latest rise time (*M* = 9:14 AM, *SD* = 1 h and 42 min), which was consistent with survey and daily diary results. Latinx students had the earliest rise time (*M* = 8:53 AM, *SD* = 2 h and 0 min), which was consistent with daily diary data.

Total time in bed was only collected in the survey and daily diaries. Significant differences in total time in bed emerged only from survey data. White students reported the longest total time in bed (*M* = 7 h and 58 min, *SD* = 1.21), while Black students reported the shortest (*M* = 7 h and 10 min, *SD* = 1.54). Asian, Latinx, and multiracial students fell in between (*M* range = 7 h and 21 min—7 h and 34 min, *SD* = 1.27– 1.56). No significant differences were observed in daily diary measures.

## Environmental Characteristics (Sleep Environment Index)

Environmental sleep disturbances were only collected in the survey and daily diaries. White and multiracial students reported significantly more environmental disturbances (*M* = 2.42 for both groups, *SD* = 1.45—1.71) compared to all other ethnic/racial groups (*M* = 1.95—2.05, *SD* = 1.52—1.65). Similar results were observed in the daily diary data, with White students reporting the most environmental sleep disturbances (*M* = 0.80, *SD* = 1.17), significantly greater than Black students, who reported the least disturbances (*M* = 0.44, *SD* = 0.81).

## Daytime Characteristics (Daytime Dysfunction)

Daytime dysfunction was only collected in the survey and daily diaries. Survey data indicated that Black, Latinx, and multiracial students experienced significantly greater daytime dysfunction (*M* = 2.88—3.00, *SD* = 1.54—1.61) compared to Asian and White students (*M* = 2.65—2.66, *SD* = 1.41—1.50). However, similar differences were not observed in the daily diary reports.

## Summary of Results

Despite differences across the survey, daily diary, and wrist actigraphy methodologies, certain sleep disparity patterns were consistent. For example, all three methods converged on finding that White students had the longest sleep duration, and across all three measures, these differences were significantly longer than those of Black students who had the shortest sleep duration. Looking at some of the other sleep metrics, it is possible that longer sleep duration for White students may be driven by later rise times found in both survey and daily diary reports and longer time in bed. At the same time, Black students reported having the latest bedtimes, sleep onset, and shortest time in bed, factors that might also explain shorter sleep duration. Black students also reported significantly higher levels of daytime dysfunction (higher than Asian and White, but not different from Latinx or multiracials), likely related to having the shortest sleep duration. Significant differences in duration were especially surprising when considered in the context that White (and multiracial) reported the most sleep environment disturbances in both surveys and daily diaries. Specifically, across both methods, White students had significantly more environmental sleep disturbances compared to Black students.

Although Asian students’sleep duration was not especially long or short relative to other groups, their sleep quality was notably worse than other groups. Specifically, actigraph data revealed Asian students to have the lowest levels of sleep efficiency, the highest level of WASO, and the highest level of sleep inertia. These disparities in sleep quality may explain why Asian students also report the shortest sleep onset latency due to fatigue, the second longest duration (after White students but not different from multiracial students), and the second latest rise time (after White students).

As expected based on prior research on sleep disparities [34, 43], Latinx and multiracial students showed some disparities, but not consistent patterns. For example, Latinx students reported the second shortest sleep duration (significantly longer than Black students, but significantly shorter than all other groups) in their surveys. They also reported falling asleep significantly faster than multiracial students, a tendency that may be related to relatively short sleep duration. Finally, Latinx students also reported high levels of daytime dysfunction, levels that were statistically indistinguishable from Black and multiracial students whose levels were significantly higher than Asian and White students. The ability to include multiracial students as a separate group is a strength of the study. Doing so revealed noteworthy disparity patterns. While multiracial students reported the second longest sleep duration in their surveys and actigraphy (statistically indistinguishable from Asians, but significantly shorter than White students), they also had significantly more environmental sleep disruptors and the highest levels of daytime dysfunction (statistically indistinguishable from Black and Latinx, but significantly higher than Asian and White students).

## Discussion

The goal of this study was to examine ethnic/racial sleep disparities in a diverse sample of first-year college students using a combination of survey, daily diary, and actigraph methodologies. In line with our hypothesis and existing data, ethnic/racial disparities exist during the first semester of college. Not surprisingly, White students overall report more favorable sleep outcomes compared to ethnic/racial minorities particularly related to sleep duration and related indices (i.e., rise time, in-bed time). In contrast, Black students tended to exhibit the most prevalent and consistent disparities compared to other groups, with Asian, Latinx, and multiracial students falling in between. These findings are consistent with previous studies that also document similar sleep patterns across ethnic/racial groups in the United States [15, 16, 44]. What the current study adds is an investigation of disparities at the critical period of college students’ first college semester. Because we included multiple indices of sleep across three complementary methodologies, the current study also offers unique insight into what may be driving observed disparities. For example, it seems possible that the benefits White students have in longer sleep duration are driven by other sleep-related behaviors such as rise times and in-bed times. Moreover, because we saw convergence across surveys, daily diaries, and actigraphy, we have converging confidence concluding that White students do in fact sleep significantly longer than students from other groups. In addition, this observation is underscored despite the fact that White students report the highest levels of sleep-related environmental disturbances (i.e., noise, room temperature, and light). This unexpected finding is worthy of future investigation and may suggest that the true magnitude of disparities is actually larger than our data suggest.

By the same token, the observed disparities for Black students were also largely consistent with existing research [34]. Black students reported the latest bedtimes and the shortest sleep duration, despite reporting significantly fewer environmental sleep disturbances than White students. These findings suggest that self-reported environmental factors (at least as they were measured in this study) may not be driving these disparities. As found in other research, social determinants may be more important, such as psychological experiences (i.e., stress, rumination, hypervigilance, discrimination), which have been found to disproportionately impact ethnic/racial minorities. [17, 45–47]. For example, experiences of discrimination have been found to mediate the association between Black college students reporting greater increases in sleep problems compared to White students [17]. Findings such as this highlight how experiences of discrimination contribute to ethnic/racial disparities in sleep health [17]. Especially in the context of a predominantly White institution, greater social stress among ethnically and racially minoritized college students (i.e., ethnic/racial discrimination, lack of belonging [48, 49]), financial stress [35], or academic pressures (i.e., imposter phenomenon, or feeling fraudulent while downplaying personal achievement [49]) may have more profound effects. These possibilities are supported by the observation that Black students in the current sample also reported the lowest levels of sleep quality and among the highest levels of daytime dysfunction. Taken together, it is clear that Black students suffer sleep disparities in the areas of duration, quality, and sleep–wake associations (i.e., daytime consequences of poor sleep).

For Asian students, disparities are a bit more mixed, consistent with prior research on adolescent sleep [15]. Asians reported shorter sleep durations than White students, but significantly longer sleep durations than Black and Latinx students, which aligns with previous findings [15, 16, 44, 50, 51]. Interestingly, Asian students showed notable disparities in sleep quality with actigraphy data capturing the lowest sleep efficiency, greatest WASO, and greatest sleep inertia. It is likely that Asian students’ poor quality of sleep throughout the night explains why they take more time getting out of bed after waking up. This finding is consistent with previous research that poorer sleep quality increases the risk of experiencing sleep inertia among college students [52]. Although there is limited research on sleep quality among Asian college students, one study that included Asian, Black, Latinx, and White students found that only Asian (but not Black and Latinx) students, reported significantly greater nonrestorative sleep (i.e., waking up feeling unrested) relative to White students [19]. The current study contributes to this body of evidence by providing actigraphy-based results that corroborate previous self-reported findings [19]. However, the drivers of poorer sleep quality among Asian students relative to the other ethnically and racially minoritized groups, compared to White participants, remains unclear and is an area ripe for future research.

The sleep patterns observed among Latinx students suggest disparities related to duration and quality. Latinx students reported the second shortest sleep duration, significantly longer than only Black students. They also reported the highest levels of daytime dysfunction (i.e., levels that were statistically indistinguishable from Black and multiracial students and significantly higher than Asian and White students) and took the longest to fall asleep. Although Asian students reported the worst sleep quality, these comparisons were relative to Latinx students where they have relatively earlier bedtimes and later wake times. There is limited research on why this pattern was found, although one study on Latinx parents by Martinez et al. (2015) found that consistent bedtime routines were a common theme when discussing factors related to achieving optimal sleep duration for their children [53]. Thus, parental socialization around sleep norms that prioritize routines may be a contributing factor for Latinx college students, although future research should explore other variables such as academic or cultural factors.

A key contribution of this research is the systematic inclusion of multiracial students, a group that is often too small to include analytically. Because there is so little research on this group, we were not sure what to expect in terms of sleep disparities. Of note, this group is heterogeneous in terms of ethnicity and race (e.g., Latinx and Black, Latinx and White, Asian and White), therefore the mixed findings may not be too surprising. Multiracial students had the second highest level of sleep duration according to survey and actigraphy results, but they also took significantly longer to fall asleep compared to Latinx students. For sleep quality, actigraphy data showed that multiracial students had significantly better sleep efficiency, fewer WASO, and less sleep inertia relative to Asian students. However, multiracial students also exhibited areas of vulnerability related to reported one of the highest levels of environmental sleep disruptors and daytime dysfunction. Taken together, these findings contribute to existing research on sleep disparities with multiracial children [43]. Wang et al. (2024) found that multiracial and Asian children exhibited disparities in sleep when compared to Latinx children (e.g., less sleep efficiency) [43]. In contrast, this study found that multiracial and Latinx students exhibited disparities when compared to Asian children (e.g., greater sleep efficiency, less WASO, and less sleep inertia). Additionally, previous research on adolescents has found multiracial students to have poorer sleep outcomes compared to monoracial students [54]. Notably, Goodines et al. (2020) also found that discrimination towards multiracial adolescents played a mediating role in linking multiracial status and poor sleep [54]. It is possible that discrimination experiences for multiracial individuals may differ in a college context compared to high school [55]. Additionally, multiracial students represent a very heterogeneous group, and discrimination experiences may also differ across different individuals of multiracial status [19, 56]. Nevertheless, these findings support the ongoing effort in considering multiracial status in sleep disparity research [43, 54, 57].

## Comparisons to National Recommendations

Regardless of ethnic/racial group membership, students in their first semester of college met the nationally recommended threshold of 7–9 h based on actigraph data (range: 7 h and 1 min—7 h and 28 min [32]). Daily diary data generally supported these findings (range: 7 h and 1 min—7 h and 14 min), with the exception of Black students, who reported 12 min below the national threshold (*M* = 6 h and 48 min). However, these patterns differed from students’ survey reports, which suggested that all groups slept below the nationally recommended minimum threshold (between “5–6” and “6–7 h”). Additionally, it is worth noting that there was more variability around sleep duration means for ethnically and racially minoritized students than there was for White students (Table 3).

In terms of sleep quality, all students experienced poorer sleep quality than the standard thresholds. All ethnic/racial groups experienced low sleep efficiency (< 85%; range: 80.97%—82.77% [58]), which may be driven by higher WASO (> 20 min; range: 43.27—49.71 min [59]). Although first-year college students may be getting *sufficient* sleep, the *quality* of their sleep remains compromised, potentially diminishing the restorative benefits of sleep [58, 59].

## Methods for Measuring Sleep

Another contribution of this study is the use of multiple sleep indicators. For the most part, disparities were observed in the survey measures, but not as consistently in the daily diary or actigraphy data. Since survey and daily diary data are both self-reported, one would expect that these two data sources would be more similar due to a mono-method bias, however, this did not seem to be the case. Similarly, since daily diary and actigraphy were measured on the same nights, one might also expect these two to converge, however, this was also not the case. It is possible that certain sleep dimensions such as quality, latency, or daytime dysfunction, aspects of sleep that may be more variable over time, may be better suited to intensive, repeated assessments. However, variables such as sleep environment may not need to be measured on a repeated basis.

## Limitations, Implications, and Future Directions

A primary limitation of the current study is that the sample consisted of first-year, first-semester undergraduate students enrolled at one university, limiting the generalizability of these findings. This is particularly worth noting since this university is a private, predominantly White university in a large and diverse urban area. As such, the current sample may have unique student experiences that may influence sleep patterns, such as increased urbanicity (i.e., greater noise/light exposure throughout the day) and commuting [60, 61]. Thus, more research is needed to investigate ethnic/racial disparities in sleep over time in more generalized settings, such as in public universities or in less urban areas. Expanding these findings across diverse educational contexts can help elucidate the generalizability of ethnic/racial sleep disparities across different populations.

Nevertheless, this study underscores the importance of investigating ethnic/racial sleep disparities, particularly during the first semester of college, a critical period for establishing health behaviors that can have long-term impacts on academic and mental health outcomes [1]. Future research should investigate whether emerging adults come to college with already compromised sleep, or if the college context itself produces sleep problems. First-year college students may be getting sufficient sleep but suffering from compromised sleep quality, emphasizing the need for colleges to promote early sleep interventions for college students that focus on getting restful sleep [9]. Additionally, the relative disadvantages in sleep duration for Black students, sleep quality for Asian students, and the nuanced challenges of Latinx and multiracial students highlight the need for targeted campus interventions that focus on supporting ethnically/racially minoritized students. In particular, these disparities do not seem to stem from environmental disturbances, suggesting that future research on ethnical/racial disparities in college student sleep should focus on psychological factors, such as stress, rumination, or hypervigilance [45–47]. Addressing these disparities through culturally responsive programs, increased mental health support, and campus resources that foster more restful sleep could help mitigate the negative effects of poor sleep and promote overall well-being and academic success for new college students.

## Figures and Tables

**Table 1:** Gender differences in sleep outcomes

	Female	Male	Non-binary	F-test	Contrasts

*Survey*	*M (SD)*	*M (SD)*	*M (SD)*		
Sleep latency	.89 (.86)	.83 (.78)	1.63 (.76)	29.65***	MA < F < NB
Sleep duration	1.74 (.83)	1.86 (.87)	1.45 (.92)	15.11***	NB < F < MA
Sleep quality	1.86 (.58)	1.96 (.50)	1.59 (.90)	23.25***	NB < F < MA
Bedtime	01:02 AM (1 h 0 min)	00:22 AM (1 h 17 min)	02:07 AM (1 h 4 min)	9.81***	MA < F < NB
Rise time	08:00 AM (1 h 17 min)	08:13 AM (1 h 27 min)	08:14 AM (1 h 0 min)	12.45***	F < MA
Sleep environment	2.13 (1.58)	2.23 (1.58)	3.22 (1.08)	30.40***	F, MA < NB
Daytime dysfunction	2.99 (1.52)	2.35 (1.46)	3.73 (1.17)	107.2***	MA < F < NB
	Female	Male	Non-binary	*χ^2^*	Contrasts
*14-day Daily Diary*	*M (SD)*	*M (SD)*	*M (SD)*		
Sleep latency	1.25 (1.09)	1.23 (1.06)	1.77 (1.11)	10.78***	F, MA < NB
Sleep duration	8.90 (4.57)	9.03 (4.65)	7.89 (3.66)	1.97	
Sleep quality	1.99 (.75)	2.07 (.74)	1.97 (.76)	8.76***	F < MA
Bedtime	00:32 AM (1 h 0 min)	1:18 AM (1 h 50 min)	1:51 AM (1 h 55 min)	10.79***	MA < F
Rise time	08:40 AM (1 h 9 min)	08:50 AM (1 h 6 min)	08:48 AM (1 h 10 min)	3.35*	F < MA
Sleep environment	.67 (1.07)	.59 (1.01)	1.08 (1.12)	19.40***	MA < F < NB
Daytime dysfunction	.59 (.78)	.40 (.68)	.89 (.79)	35.84***	MA < F < NB
	Female	Male	Non-binary	*χ^2^*	Contrasts
*14-day Actigraph*	*M (SD)*	*M (SD)*	*M (SD)*		
Sleep latency	20.12 (24.22)	20.38 (25.69)	14.65 (23.54)	.45	
Sleep onset	01:16 AM (1 h 53 min)	01:49 AM (1 h 48 min)	01:03 AM (2 h 35 min)	34.93***	F, NB < MA
Sleep offset	08:42 AM (1 h 7 min)	0:8:55 AM (1 h 9 min)	09:05 AM (1 h 25 min)	23.78***	F < MA < NB
Sleep duration	7 h 39 min (1 h 43 min)	7 h 14 min (1 h 35 min)	7 h 42 min (1 h 24 min)	23.92***	MA < F
Sleep efficiency	82.82% (7.15)	80.64% (7.88)	79.62% (6.44)	39.05***	MA, NB < F
Wake after sleep onset (WASO)	44.75 (15.32)	49.03 (15.76)	59.97 (17.04)	54.61***	F < MA < NB
Bedtime	12:56 AM (1 h 55 min)	01:29 AM (1 h 50 min)	12:39 AM (2 h 33 min)	33.32***	F, NB < MA
Rise time	09:00 AM (2 h 0 min)	09:14 AM (2 h 5 min)	09:26 AM (2 h 25 min)	8.32***	F < MA

F = female, MA = male, NB = non-binary M = mean, SD = standard deviation h = hour, min = minute

* *p* <.05,

** *p* <.01,

*** p <.001

**Table 2:** Socioeconomic differences in sleep outcomes

	Pell recipient	Did not receive Pell	F-test	Contrasts

*Survey*	*M (SD)*	*M (SD)*		
Sleep latency	0.86 (.86)	0.92 (.82)	2.16	
Sleep duration	1.70 (.85)	1.86 (.83)	17.49***	P < NP
Sleep quality	1.86 (.55)	1.91 (.59)	3.47*	P < NP
Bedtime	12:49 AM (1 h 47 min)	1:28 AM (2 h 2 min)	3.78*	P < NP
Rise time	07:53 AM (1 h 19 min)	08:11 AM (1 h 12 min)	36.96***	P < NP
Sleep environment	2.19 (1.66)	2.12 (1.52)	13.47***	NP < P
Daytime dysfunction	2.97 (1.50)	2.66 (1.52)	23.13***	NP < P
	Pell recipient	Did not receive Pell	*χ^2^*	Contrasts
*14-day Daily Diary*	*M (SD)*	*M (SD)*		
Sleep latency	1.25 (1.09)	1.29 (1.07)	3.18	
Sleep duration	8.99 (4.77)	8.79 (4.31)	1.47	
Sleep quality	2.04 (.74)	1.99 (.73)	2.68*	NP < P
Bedtime	01:31AM (1 h 57 min)	12:43 AM (1 h 29 min)	6.35***	NP < P
Rise time	08:29 AM (2 h 16 min)	08:57 AM (2 h 13 min)	23.77***	P < NP
Sleep environment	.64 (1.03)	.69 (1.08)	4.15**	P < NP
Daytime dysfunction	.56 (.77)	.53 (.75)	1.23	
	Pell recipient	Non-Pell recipient	*χ^2^*	Contrasts
*14-day Actigraph*	*M (SD)*	*M (SD)*		
Sleep latency	10.85 (24.42)	20.79 (25.37)	2.59	
Sleep onset	01:16 AM (1 h 54 min)	01:34 AM (1 h 48 min)	46.01***	P < NP
Sleep offset	08:35 AM (1 h 16 min)	08:59 AM (1 h 13 min)	69.85	P < NP
Sleep duration	7 h 36 min (1 h 43 min)	7 h 31 min (1 h 40 min)	4.15*	P < NP
Sleep efficiency	82.09 (7.59)	82.39 (7.40)	2.92	
Wake after sleep onset (WASO)	45.76% (26.12)	46.89% (26.28)	3.42	
Bedtime	12:55 AM (1 h 56 min)	01:14 AM (1 h 51 min)	48.9***	P < NP
Rise time	08:55 AM (1 h 58 min)	09:17 AM (2 h 4 min)	58.59***	P < NP

P = Pell recipient, NP = Did not receive Pell M = mean, SD = standard deviation h = hour, min = minute

* *p* <.05,

** *p* <.01,

*** p <.001

**Table 3:** Ethnic/racial differences in sleep outcomes

	Asian	Black	Latinx	Multiracial	White	F-test (df)	Contrasts

*Survey*	*M (SD)*	*M (SD)*	*M (SD)*	*M (SD)*	*M (SD)*		
Sleep latency	0.82 (.83)	0.92 (.88)	0.83 (.82)	0.92 (.85)	0.90 (.81)	4.28** (4, 3826)	A, L < B, MR
Sleep duration	1.73 (.86)	1.42 (.83)	1.65 (.86)	1.72 (.77)	2.00 (.77)	104.08*** (4, 3788)	B, L, A, MR < W B, L < A, MR B < L
Sleep quality	1.92 (.50)	1.80 (.65)	1.89 (.57)	1.90 (.54)	1.89 (.56)	7.74*** (4, 3763)	B < A, L, MR, W A < L, W
Bedtime	00:27 AM (1 h 15 min)	00:38 AM (1 h 27 min)	00:23 AM (1 h 15 min)	00:23 AM (1 h 13 min)	00:22 AM (1 h 5 min)	0.69 (4, 3774)	
Rise time	08:06 AM (1 h 26 min)	07:50 AM (1 h 26 min)	07:56 AM (1 h 19 min)	07:55 AM (1 h 11 min)	08:20 AM (1 h 14 min)	40.60*** (4, 3532)	B, L, MR, A < W B, L, MR < A
Total time in bed	7 h 34 min (1.56)	7 h 10 min (1.54)	7 h 31 min (1.49)	7 h 27 min (1.27)	7 h 58 min (1.21)	72.32*** (4, 3502)	B, L, MR, A < W B < L, MR, A MR < A
Sleep environment	2.05 (1.65)	2.01 (1.56)	1.95 (1.52)	2.42 (1.71)	2.42 (1.45)	37.76*** (4, 3795)	A, B, L < MR, W
Daytime dysfunction	2.66 (1.50)	2.88 (1.61)	3.00 (1.60)	2.88 (1.54)	2.65 (1.41)	18.00*** (4, 3778)	W, A < B, L, MR MR < L
	Asian	Black	Latinx	Multiracial	White	*χ^2^*	Contrasts
*14-day Daily Diary*	*M (SD)*	*M (SD)*	*M (SD)*	*M (SD)*	*M (SD)*		
Sleep latency	1.22 (1.08)	1.25 (1.04)	1.13 (1.07)	1.29 (1.10)	1.33 (1.08)	0.46	
Sleep duration	7 h 1 min (1 h 45 min)	6 h 48 min (1 h 49 min)	7 h 9 min (1 h 49 min)	7 h 0 min (1 h 51 min)	7 h 14 min (1 h 41 min)	13.97**	B < L, W
Sleep quality	2.02 (.73)	2.06 (.73)	2.01 (.74)	2.00 (.77)	2.01 (.75)	0.05	
Bedtime	01:24 AM (3 h 59 min)	01:27 AM (3 h 50 min)	00:49 AM (4 h 24 min)	01:18 AM (4 h 23 min)	01:12 AM (4 h 2 min)	4.53***	L < A, B, W
Rise time	08:37 AM (2 h 14 min)	08:22 AM (2 h 22 min)	08:21 AM (2 h 0 min)	08:34 AM (2 h 19 min)	08:58 AM (1 h 58 min)	25.63***	B, L, MR < W
Total time in bed	8 h 49 min (4 h 34 min)	8 h 20 min (4 h 26 min)	8 h 53 min (4 h 38 min)	9 h 5 min (4 h 47 min)	9 h 2 min (4 h 22 min)	6.10	
Sleep environment	0.56 (1.00)	0.43 (.81)	0.60 (1.01)	0.70 (1.11)	0.79 (1.17)	15.51**	B < W
Daytime dysfunction	0.49 (.74)	0.54 (.78)	0.58 (.79)	0.55 (.76)	0.49 (.74)	4.35	
	Asian	Black	Latinx	Multiracial	White	*χ^2^*	Contrasts
*14-day Actigraph*	*M (SD)*	*M (SD)*	*M (SD)*	*M (SD)*	*M (SD)*		
Sleep latency	21.20 min (25.85)	18.18 min (22.86)	21.18 min (25.63)	21.32 min (26.46)	19.43 min (24.35)	15.29***	B < W < MR, L B < A < L
Sleep onset	01:20 AM (1 h 52 min)	01:38 AM (2 h 3 min)	01:15 AM (1 h 54 min)	01:22 AM (1 h 54 min)	01:25 AM (1 h 41 min)	6.59***	A, L, MR, W < B
Sleep offset	08:41 AM (2 h 6 min)	08:40 AM (2 h 5 min)	08:34 AM (1 h 57 min)	08:46 AM (2 h 7 min)	08:55 AM (1 h 46 min)	0.70	
Sleep duration	7 h 19 min (1 h 56 min)	7 h 1 min (2 h 1 min)	7h13 min (1 h 59 min)	7 h 24 min (2 h 1 min)	7 h 28 min (1 h 42 min)	9.35**	B < A, L, MR, W
Sleep efficiency	80.97% (8.31)	81.97% (8.10)	82.77% (7.31)	82.67% (7.36)	82.32% (6.95)	8.41**	A < L, MR, W
Wake after sleep onset (WASO)	49.71 (28.29)	44.88 (26.55)	43.27 (26.75)	44.32 (24.19)	48.43 (25.65)	15.29**	B, L, MR < A
Sleep inertia	22.12 (26.51)	19.19 (23.08)	17.89 (20.60)	18.67 (22.42)	18.50 (20.43)	4.35**	L, MR, W < A
Bedtime	00:59 AM (1 h 53 min)	01:20 AM (2 h 3 min)	00:56 AM (1 h 54 min)	00:59 AM (1 h 54 min)	01:05 AM (1 h 42 min)	8.00***	B < A, L, MR, W
Rise time	09:03 AM (2 h 10 min)	08:57 AM (2 h 4 min)	08:52 AM (2 h 0 min)	9:04 AM (2 h 9 min)	9:13 AM (1 h 47 min)	0.51	

A = Asian, B = Black, L = Latinx, MR = multiracial, W = White; M = mean, SD = standard deviation; h = hour, min = minute

* *p* <.05,

** *p* <.01,

*** p <.001

## Data Availability

All data and code are available upon request.

## References

[1] Arnett JJ. College students as emerging adults: The developmental implications of the college context. Emerg Adulthood. 2016;4(3):219–22. 10.1177/2167696815587422.

[2] Becerra MB, Becerra BJ. Psychological Distress among College Students: Role of Food Insecurity and Other Social Determinants of Mental Health. Int J Environ Res Public Health. 2020;17(11):4118. 10.3390/ijerph17114118. PMC7312727 32526990

[3] Wang F, Bíró É. Determinants of sleep quality in college students: A literature review. Explore (NY). 2021;17(2):170–7. 10.1016/j.explore.2020.11.003. 33246805

[4] Smout S, Champion KE, O’Dean S, Halladay J, Gardner LA, Newton NC. Adolescent lifestyle behaviour modification and mental health: Longitudinal changes in diet, physical activity, sleep, screen time, smoking, and alcohol use and associations with psychological distress. International Journal of Mental Health and Addiction. 2024:Advance online publication. 10.1007/s11469-024-01350-9

[5] Atoui S, Chevance G, Romain AJ, Kingsbury C, Lachance JP, Bernard P. Daily associations between sleep and physical activity: A systematic review and meta-analysis. Sleep Med Rev. 2021;57: 101426. 10.1016/j.smrv.2021.101426. 33571893

[6] Lev-Ari L, Shmuel S. Pathways of sleep, affect, and stress constellations during the first year of college: Transition difficulties of emerging adults. J Youth Stud. 2011;15(3):273–92. 10.1080/13676261.2011.635196.

[7] Lund HG, Reider BD, Whiting AB, Prichard JR. Sleep patterns and predictors of disturbed sleep in a large population of college students. J Adolesc Health. 2010;46(2):124–32. 10.1016/j.jadohealth.2009.06.016. 20113918

[8] Becker SP, Jarrett MA, Luebbe AM, Garner AA, Burns GL, Kofler MJ. Sleep in a large, multi-university sample of college students: sleep problem prevalence, sex differences, and mental health correlates. Sleep Health: J Natl Sleep Found. 2018;4(2):174–81. 10.1016/j.sleh.2018.01.001. PMC5863586 29555131

[9] Hershner S, O’Brien LM. The Impact of a Randomized Sleep Education Intervention for College Students. J Clin Sleep Med. 2018;14(03):337–47. 10.5664/jcsm.6974. PMC5837835 29510791

[10] Gardani M, Bradford DRR, Russell K, A systematic review and meta-analysis of poor sleep, insomnia symptoms and stress in undergraduate students. Sleep Med Rev. 2022;61: 101565. 10.1016/j.smrv.2021.101565. 34922108

[11] Lunsford-Avery JR, Wang KW, Kollins SH, Chung RJ, Keller C, Engelhard MM. Regularity and Timing of Sleep Patterns and Behavioral Health Among Adolescents. J Dev Behav Pedia: JDBP. 2022;43(4):188–96. 10.1097/DBP.0000000000001013. PMC9035469 34698705

[12] Chung J, Goodman M, Huang T, Bertisch S, Redline S. Multidimensional sleep health in a diverse, aging adult cohort: Concepts, advances, and implications for research and intervention. Sleep Health. 2021;7(6):699–707. 10.1016/j.sleh.2021.08.005. PMC8665047 34642124

[13] Jackson CL, Walker JR, Brown MK, Das R, Jones NL. A workshop report on the causes and consequences of sleep health disparities. Sleep. 2020;43(8):zsaa037. 10.1093/sleep/zsaa037. PMC7420527 32154560

[14] Fuller-Rowell TE, Curtis DS, El-Sheikh M, Duke AM, Ryff CD, Zgierska AE. Racial discrimination mediates race differences in sleep problems: A longitudinal analysis. Cultur Divers Ethnic Minor Psychol. 2017;23(2):165–73. 10.1037/cdp0000104. PMC5243865 27429065

[15] Guglielmo D, Gazmararian JA, Chung J, Rogers AE, Hale L. Racial/ethnic sleep disparities in US school-aged children and adolescents: a review of the literature. Sleep Health. 2018;4(1):68–80. 10.1016/j.sleh.2017.09.005. PMC5771439 29332684

[16] Yip T, Cheon YM, Wang Y, Cham H, Tryon W, El-Sheikh M. Racial Disparities in Sleep: Associations With Discrimination Among Ethnic/Racial Minority Adolescents. Child Dev. 2020;91(3):914–31. 10.1111/cdev.13234. PMC11174141 30942498

[17] Bermudez VN, Fearon-Drake D, Wheelis M, Cohenour M, Suntai Z, Scullin MK. Sleep disparities in the first month of college: implications for academic achievement. Sleep Adv J Sleep Res Soc. 2022;3(1):zpac041. 10.1093/sleepadvances/zpac041. PMC10104382 37193411

[18] Dzierzewski JM, Ravyts SG, Dautovich ND, Perez E, Schreiber D, Rybarczyk BD. Mental health and sleep disparities in an urban college sample: A longitudinal examination of White and Black students. J Clin Psychol. 2020;76(10):1972–83. 10.1002/jclp.22974. PMC7487046 32410237

[19] Jones RD, Jackson WB 2nd, Mazzei A, Chang AM, Buxton OM, Jackson CL. Ethnoracial sleep disparities among college students living in dormitories in the United States: a nationally representative study. Sleep Health. 2020;6(1):40–7. 10.1016/j.sleh.2019.10.005. PMC6995403 31759933

[20] Gaultney JF. The prevalence of sleep disorders in college students: impact on academic performance. J Am Coll Health. 2010;59(2):91–7. 10.1080/07448481.2010.483708. 20864434

[21] Goodin BR, McGuire L, Smith MT. Ethnicity Moderates the Influence of Perceived Social Status on Subjective Sleep Quality. Behav Sleep Med. 2010;8(4):194–206. 10.1080/15402002.2010.509193. 20924833

[22] Carey RM, Stephens NM, Townsend SSM, Hamedani MG. Is diversity enough? Cross-race and cross-class interactions in college occur less often than expected, but benefit members of lower status groups when they occur. J Pers Soc Psychol. 2022;123(5):889–908. 10.1037/pspa0000302. 35254855

[23] Hoopes EK, Berube FR, D’Agata MN, Sleep duration regularity, but not sleep duration, is associated with microvascular function in college students. Sleep. 2021;44(2). 10.1093/sleep/zsaa175 PMC7879418 32905591

[24] Buysse DJ, Reynolds CF 3rd, Monk TH, Berman SR, Kupfer DJ. The Pittsburgh Sleep Quality Index: a new instrument for psychiatric practice and research. Psychiatry Res. 1989;28(2):193–213. 10.1016/0165-1781(89)90047-4. 2748771

[25] Ibáñez V, Silva J, Cauli O. A survey on sleep assessment methods. PeerJ. 2018;6: e4849. 10.7717/peerj.4849. PMC5971842 29844990

[26] Beatty DL, Hall MH, Kamarck TA, Unfair treatment is associated with poor sleep in African American and Caucasian adults: Pittsburgh SleepSCORE project. Health Psychol. 2011;30(3):351–9. 10.1037/a0022976. PMC3131074 21553979

[27] Yip T The effects of ethnic/racial discrimination and sleep quality on depressive symptoms and self-esteem trajectories among diverse adolescents. J Youth Adolesc. 2015;44(2):419–30. 10.1007/s10964-014-0123-x. PMC4574503 24682960

[28] Mallinson DC, Kamenetsky ME, Hagen EW, Peppard PE. Subjective sleep measurement: comparing sleep diary to questionnaire. Nat Sci Sleep. 2019;11:197–206. 10.2147/nss.S217867. PMC6752706 31686932

[29] Ancoli-Israel S, Cole R, Alessi C, Chambers M, Moorcroft W, Pollak CP. The role of actigraphy in the study of sleep and circadian rhythms. Sleep. 2003;26(3):342–92. 10.1093/sleep/26.3.342. 12749557

[30] Meltzer LJ, Montgomery-Downs HE, Insana SP, Walsh CM. Use of actigraphy for assessment in pediatric sleep research. Sleep Med Rev. 2012;16(5):463–75. 10.1016/j.smrv.2011.10.002. PMC3445439 22424706

[31] Marino M, Li Y, Rueschman MN, Measuring sleep: accuracy, sensitivity, and specificity of wrist actigraphy compared to polysomnography. Sleep. 2013;36(11):1747–55. 10.5665/sleep.3142. PMC3792393 24179309

[32] Hirshkowitz M, Whiton K, Albert SM, National Sleep Foundation’s sleep time duration recommendations: methodology and results summary. Sleep Health. 2015;1(1):40–3. 10.1016/j.sleh.2014.12.010. 29073412

[33] Jean-Louis G, Grandner MA, Seixas AA. Social determinants and health disparities affecting sleep. Lancet Neurol. 2022;21(10):864–5. 10.1016/s1474-4422(22)00347-7. 36115351

[34] Johnson DA, Lewis TT, Guo N, Associations between everyday discrimination and sleep quality and duration among African-Americans over time in the Jackson Heart Study. Sleep. 2021;44(12). 10.1093/sleep/zsab162 PMC8664593 34197610

[35] Mezick EJ, Matthews KA, Hall M, Influence of race and socioeconomic status on sleep: Pittsburgh SleepSCORE project. Psychosom Med. 2008;70(4):410–6. 10.1097/PSY.0b013e31816fdf21. PMC2887747 18480189

[36] Bagley EJ, Kelly RJ, Buckhalt JA, El-Sheikh M. What keeps low-SES children from sleeping well: the role of presleep worries and sleep environment. Sleep Med. 2015;16(4):496–502. 10.1016/j.sleep.2014.10.008. PMC4395518 25701537

[37] Henríquez-Beltrán M, Benítez ID, Vaca R, The trajectory of sleep after critical illness: a 24-month follow-up study. Ann Intensive Care. 2025;15:28. 10.1186/s13613-025-01449-9. PMC11871202 40019644

[38] Sadeh A The role and validity of actigraphy in sleep medicine: an update. Sleep Med Rev. 2011;15(4):259–67. 10.1016/j.smrv.2010.10.001. 21237680

[39] A Language and Environment for Statistical Computing. R Foundation for Statistical Computing; 2021. https://www.R-project.org/

[40] Carnethon MR, De Chavez PJ, Zee PC, Disparities in sleep characteristics by race/ethnicity in a population-based sample: Chicago Area Sleep Study. Sleep Med. 2015;18:50–5. 10.1016/j.sleep.2015.07.005. PMC4728038 26459680

[41] Welch BL. On the Comparison of Several Mean Values: An Alternative Approach. Biometrika. 1951;38(3/4):330–6. 10.2307/2332579.

[42] Bates D, Mächler M, Bolker B, Walker S. Fitting Linear Mixed-Effects Models Using lme4. J Statis Software. 2015;67(1):1–48. 10.18637/jss.v067.i01.

[43] Wang Y, Yang L, Lin G, The efficacy of progressive muscle relaxation training on cancer-related fatigue and quality of life in patients with cancer: A systematic review and meta-analysis of randomized controlled studies. Int J Nurs Stud. 2024;152: 104694. 10.1016/j.ijnurstu.2024.104694. 38281450

[44] Lorenzo K, Xie M, Cham H, El-Sheikh M, Yip T. Corresponding Changes in Sleep and Discrimination: A Three-year Longitudinal Study Among Ethnically/Racially Diverse Adolescents. J Youth Adolesc. 2024;54(2):368–82. 10.1007/s10964-024-02086-4. PMC13459717 39298096

[45] Kingsbury JH, Gibbons FX, Gerrard M. The effects of social and health consequence framing on heavy drinking intentions among college students. Br J Health Psychol. 2015;20(1):212–20. 10.1111/bjhp.12100. 24761786

[46] Slopen N, Chen Y, Guida JL, Albert MA, Williams DR. Positive childhood experiences and ideal cardiovascular health in midlife: Associations and mediators. Prev Med. 2017;97:72–9. 10.1016/j.ypmed.2017.01.002. PMC5430499 28087467

[47] Wang Y, Yip T. Parallel changes in ethnic/racial discrimination and identity in high school. J Youth Adolesc. 2020;49(7):1517–30. 10.1007/s10964-019-01186-w. PMC11200270 31938996

[48] Gordon AM, Prather AA, Dover T, Espino-Pérez K, Small P, Major B. Anticipated and Experienced Ethnic/Racial Discrimination and Sleep: A Longitudinal Study. Pers Soc Psychol Bull. 2020;46(12):1724–35. 10.1177/0146167220928859. 32571161

[49] Doughty KN, Martin-Parchment M. Imposter phenomenon and experiences of discrimination among students at a predominantly White institution. J Am Coll Health. 2025;73(1):31–5. 10.1080/07448481.2023.2198021. 37053586

[50] MarczykOrganek KD, Taylor DJ, Petrie T, Adolescent sleep disparities: sex and racial/ethnic differences. Sleep Health. 2015;1(1):36–9. 10.1016/j.sleh.2014.12.003. 29073411

[51] Singh A, Subhashini N, Sharma S, Mallick BN. Involvement of the α1-adrenoceptor in sleep-waking and sleep loss-induced anxiety behavior in zebrafish. Neuroscience. 2013;245:136–47. 10.1016/j.neuroscience.2013.04.026. 23618759

[52] Ma Z, Tao Y, Chen H, An Exploration of Self-Reported Sleep Inertia Symptoms Using Network Analysis. Nat Sci Sleep. 2022;14:661–74. 10.2147/nss.S347419. PMC9018210 35450224

[53] Martinez O, Wu E, Sandfort T, Evaluating the impact of immigration policies on health status among undocumented immigrants: a systematic review. J Immigr Minor Health. 2015;17(3):947–70. 10.1007/s10903-013-9968-4. PMC4074451 24375382

[54] Goodhines PA, LaRowe LR, Gellis LA, Ditre JW, Park A. Sleep-Related Cannabis Expectancy Questionnaire (SR-CEQ): Initial Development among College Students. J Psychoactive Drugs. 2020;52(5):401–11. 10.1080/02791072.2020.1800151. 32772641

[55] Toro JD, Hughes D. Trajectories of discrimination across the college years: Associations with academic, psychological, and physical adjustment outcomes. J Youth Adolesc. 2020;49(4):772–89. 10.1007/s10964-019-01147-3. 31650443

[56] Norman JB, Franco MG, Chen JM. Multiracial individuals’ experiences of rejection and acceptance from different racial groups and implications for life satisfaction. J Soc Psychol. 2021;163(4):459–79. 10.1080/00224545.2021.1996322. 34843426

[57] Davenport MA, Berkley S, Phillips SR, Association of Exposure to Interpersonal Racism and Racial Disparities in Inadequate Sleep Risk. J Pedia. 2025;276:114378. 10.1016/j.jpeds.2024.114378. PMC11645191 39447725

[58] Ohayon M, Wickwire EM, Hirshkowitz M, National Sleep Foundation’s sleep quality recommendations: first report. Sleep Health. 2017;3(1):6–19. 10.1016/j.sleh.2016.11.006. 28346153

[59] Buysse DJ, Ancoli-Israel S, Edinger JD, Lichstein KL, Morin CM. Recommendations for a standard research assessment of insomnia. Sleep. 2006;29(9):1155–73. 10.1093/sleep/29.9.1155. 17040003

[60] Hori D, Sasahara S, Oi Y, Relationships between insomnia, long working hours, and long commuting time among public school teachers in Japan: a nationwide cross-sectional diary study. Sleep Med. 2020;75:62–72. 10.1016/j.sleep.2019.09.017. 32853920

[61] Paksarian D, Rudolph KE, He JP, Merikangas KR. School Start Time and Adolescent Sleep Patterns: Results From the U.S. National Comorbidity Survey-Adolescent Supplement. Am J Public Health. 2015;105(7):1351–7. 10.2105/ajph.2015.302619. PMC4463387 25973803

